# Signals of value drive engagement with multi-round information interventions

**DOI:** 10.1371/journal.pone.0276072

**Published:** 2022-10-25

**Authors:** Jessica Lasky-Fink, Todd Rogers

**Affiliations:** 1 Goldman School of Public Policy, University of California, Berkeley, Berkeley, California, United States of America; 2 Harvard Kennedy School, Harvard University, Cambridge, Massachusetts, United States of America; Carnegie Mellon Univeristy, UNITED STATES

## Abstract

For information interventions to be effective, recipients must first engage with them. We show that engagement with repeated digital information interventions is shaped by subtle and strategically controllable signals of the information’s value. In particular, recipients’ expectations are shaped by signals from the “envelope” that surrounds a message in an information intervention. The envelope conveys clues about the message but does not reveal the message itself. When people expect the message to be valuable, delivering it in a consistent and recognizable envelope over time *increases* engagement relative to varying the envelope. Conversely, when people expect the message to be of little value, delivering it in a consistent and recognizable envelope *decreases* engagement relative to varying the envelope. We show this with two field experiments involving massive open online courses and one online survey experiment (all pre-registered, *N* = 439,150).

## Introduction

Information interventions are a common and potent tool for motivating behaviors from voting to healthy eating to enrolling in government programs. Some information interventions involve just a single communication, while others may involve multiple rounds either because of non-response to earlier communications or because the intended behavior must be repeated over time. Regardless of how many rounds are administered, the efficacy of any information intervention depends on two distinct psychological stages. First, people must decide to engage with the communication. Second, conditional on that initial engagement, the communication’s message must be understood and must motivate the intended behavior. In information interventions that involve multiple rounds of communication, these stages influence the impact of each discrete round, as well as the entire intervention. In this manuscript, we examine what affects decisions to engage with repeated communications in multiple-round information interventions.

We propose that people engage with each round of communication in an information intervention based on their expectations about the value or relevance of the message it contains [1, 2]. This expected utility value is informed by the packaging or “envelope” around the message, which conveys clues about the message, but does not reveal the full message itself. For instance, in a digital information intervention, the envelope may include an email’s sender and subject line, the phone number from which a text message is sent, or the “preview” line of a text message or email. People’s expectations about the value of a message are shaped by signals from the message’s envelope. These expectations then affect whether they engage with the contained message.

The signal of any given envelope is largely idiosyncratic to the recipient-sender context, especially when a recipient encounters it for the first time. But an envelope sends a clearer and more predictable signal when a recipient has encountered it previously. In multiple-round interventions, a recipient’s expected utility value of each round’s message is informed by their experiences engaging with previous rounds. When recipients encounter the same envelope repeatedly across communication rounds, they learn to recognize the envelope and come to identify it with the expected utility value of its contained message—regardless of whether the message content itself changes across rounds.

Understanding what affects engagement with multiple-round information interventions is important because many welfare-improving behaviors require repeated action over time. For example, multiple-round information interventions have been used to increase vaccination take-up [3]; improve medication adherence [4, 5]; increase savings [6]; promote exercise [7]; improve student achievement [8] and attendance [9, 10]; and support employee engagement [11] and student engagement [12].

Yet, evidence on the effectiveness of multiple-round interventions over time is mixed. Some studies have found that the impact of multiple-round interventions diminishes over time, such that later rounds have little to no impact [13–15]. Other studies have observed positive incremental effects for later rounds of intervention [16–18]. Despite their prevalence, prior research has failed to develop a theoretical framework to explain why the impact of sequential rounds of intervention varies [19]. This manuscript helps fill this gap.

In two pre-registered field experiments (*N* = 199,162; *N* = 239,125) and one pre-registered online experiment (*N* = 863), we test how an intervention’s envelope shapes people’s engagement in multiple-round digital information interventions. Study 1 tests our overarching theory by showing that when recipients expect a message to be valuable, keeping the envelope consistent across rounds increases engagement relative to varying the envelope. At the same time, when recipients expect a message to be of little value, a consistent envelope decreases engagement across rounds relative to varying the envelope. Studies 2 and 3 present evidence from two large-scale field experiments, which together demonstrate that varying the envelope of a low-value multiple-round information intervention leads to relatively higher engagement over time compared to delivering the information in a consistent envelope.

## Study 1

For a multiple-round information intervention to be effective over time, people must engage with each intervention round’s message. We hypothesized that the digital “envelope” surrounding the message—in this case, an email subject line—affects expectations about its value. Across rounds, people learn to associate the envelope with the expected value of the message content and, in turn, this affects their decision to engage (or not). Study 1 tests this hypothesis directly in an online factorial experiment.

### Methods and materials

#### Participants

We recruited 950 (M_age_ = 41 years; 43% female) participants on Amazon MTurk. We determined the sample size ex ante based on the smallest effect size of interest, *d* = 0.2. We exclude 87 responses from workers who completed the survey more than once. These responses were balanced evenly across experimental conditions (χ^2^(3) = 1.67, *p* = .64). This yields a final analytic sample of 863 workers, (M_age_ = 41 years; 44% female).

#### Procedure

In a pre-registered (https://osf.io/nwspm) online 2 (information value: high, low) x 2 (subject line text: same, varied) experiment, all participants who consented to participate and passed an initial attention check were randomly assigned to a high value or low value condition and to a same or varied subject line condition. This study was approved by Harvard University’s Committee on the Use of Human Subjects (IRB19-1141).

In total, there were a total of four experimental conditions:

1. High value, same subject line
2. High value, varying subject line
3. Low value, same subject line
4. Low value, varying subject line

In each condition, participants were asked four identically structured questions in separate stages. In each stage, participants were shown two possible subject lines that they might receive in their email inbox, and asked to make a binary choice about which to open. After making a choice, participants were first shown the content of the message they selected to open. They were then shown the content of the message they did *not* choose to open. Thereafter, they proceeded to the next stage and corresponding subject line choice set. This process was repeated three times in the “learning phase” (see Table 1). The fourth stage constituted the “outcome phase” during which participants were again given a two subject line choice set, and asked to choose one to open.

**Table 1:** Study 1 design.

	Condition			
Question	High value, same subject line	High value, varying subject line	Low value, same subject line	Low value, varying subject line
**Learning phase**				
**Stage 1:** Which of the following messages would you choose to open?	*Focal subject ($0*.*50 bonus)*: Discover new ideas	*Focal subject ($0*.*50 bonus)*: Discover new ideas	*Focal subject ($0 bonus)*: Discover new ideas	*Focal subject ($0 bonus)*: Discover new ideas
	*Non-Focal subject ($0*.*05 bonus)*: Today’s stories	*Non-Focal subject ($0*.*05 bonus)*: Today’s stories	*Non-Focal subject ($0*.*05 bonus)*: Today’s stories	*Non-Focal subject ($0*.*05 bonus)*: Today’s stories
**Stage 2:** Which of the following messages would you choose to open?	*Focal subject ($0*.*50 bonus)*: Discover new ideas	*Focal subject ($0*.*50 bonus)*: Don’t miss out	*Focal subject ($0 bonus)*: Discover new ideas	*Focal subject ($0 bonus)*: Don’t miss out
	*Non-Focal subject ($0*.*05 bonus)*: You’ll want to open this	*Non-Focal subject ($0*.*05 bonus)*: You’ll want to open this	*Non-Focal subject ($0*.*05 bonus)*: You’ll want to open this	*Non-Focal subject ($0*.*05 bonus)*: You’ll want to open this
**Stage 3:** Which of the following messages would you choose to open?	*Focal subject ($0*.*50 bonus)*: Discover new ideas	*Focal subject ($0*.*50 bonus)*: To make you smile	*Focal subject ($0 bonus)*: Discover new ideas	*Focal subject ($0 bonus)*: To make you smile
	*Non-Focal subject ($0*.*05 bonus)*: Volunteering questionnaire	*Non-Focal subject ($0*.*05 bonus)*: Volunteering questionnaire	*Non-Focal subject ($0*.*05 bonus)*: Volunteering questionnaire	*Non-Focal subject ($0*.*05 bonus)*: Volunteering questionnaire
**Outcome phase**				
**Stage 4:** Which of the following messages would you choose to open?	*Focal subject ($0*.*50 bonus)*: Discover new ideas	*Focal subject ($0*.*50 bonus)*: You can do anything	*Focal subject ($0 bonus)*: Discover new ideas	*Focal subject ($0 bonus)*: You can do anything
	*Non-Focal subject ($0*.*05 bonus)*: Our commitment to you	*Non-Focal subject ($0*.*05 bonus)*: Our commitment to you	*Non-Focal subject ($0*.*05 bonus)*: Our commitment to you	*Non-Focal subject ($0*.*05 bonus)*: Our commitment to you

As shown in Table 1, each stage contained a “focal” subject line and a “non-focal” subject line. Participants were not aware of their condition assignment and, by extension, were not aware of which subject line was their “focal” subject line.

In an incentive-compatible design, all participants were awarded monetary bonuses corresponding to the value of the email they chose to open at each stage: $0.50 for a high value message; $0.05 for a medium value message; and $0.00 for a low value message. The non-focal subject lines were different across stages, but the same across all conditions, and always contained medium value content when selected ($0.05 bonus). Focal subject lines contained either high value or low value content depending on condition assignment. Half of the participants were assigned to receive the same focal subject line for all four stages (same subject line conditions); the other half of the participants were assigned to receive a different focal subject line for each of the four stages (varying subject line conditions).

Our outcome of interest was which subject line participants selected in the fourth stage, measured as a binary indicator reflecting whether the focal subject line corresponding with condition assignment was chosen. We hypothesized that participants in the same subject line conditions would learn to recognize their focal subject line and associate it with the value of the enclosed information (as determined by their condition assignment). In turn, we predicted a higher percentage of participants in the *high value-same subject line* condition would open the focal subject line than in the *high value-varying subject line* condition, and that the converse would be true in the low value conditions.

### Results and discussion

**Fig 1:**
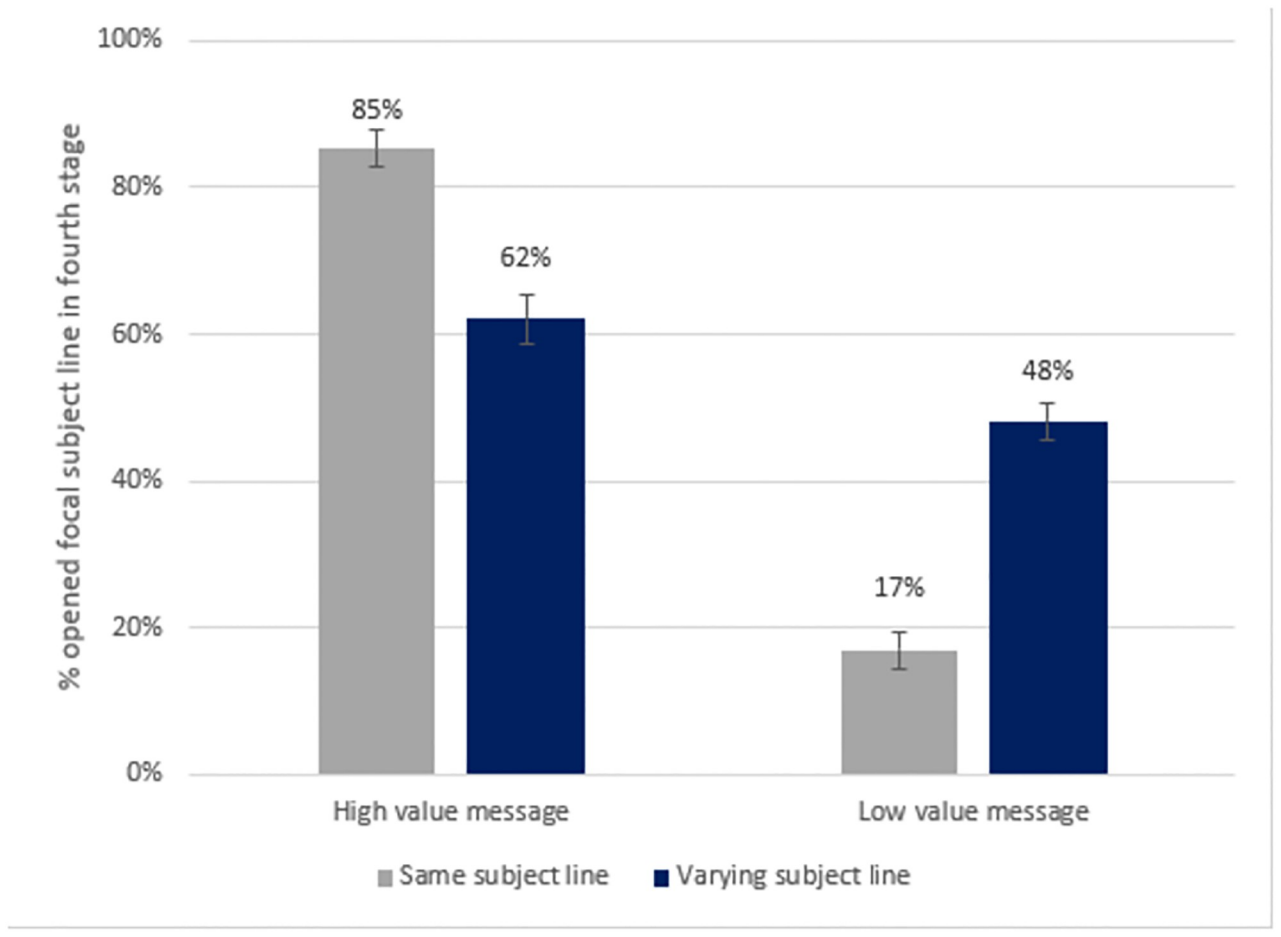
Study 1: Packaging information in the same envelope over time increases engagement with high-value messages, whereas varying the envelope increases engagement with low-value messages.

As predicted, we find that just 17% of participants in the *low value-same subject line* condition (*SE* = 2.9, 95% CI [11.0, 22.4]) opened the focal message in the fourth stage compared to 48% of participants in the *low value-varying subject line* condition (*SE* = 2.9, 95% CI [42.2, 53.7]). In contrast, 62% of participants in the *high value-varying subject line* condition (*SE* = 2.9, 95% CI [56.1, 67.8]) and 85% of participants in the *high value-same subject line* condition (*SE* = 2.9, 95% CI [79.6, 91.2]) opened the focal message in the fourth stage (see Fig 1).

Study 1 demonstrates that the envelope surrounding a digital information intervention becomes a signal for its value across repeated exposures. As a result, when the enclosed message is expected to be valuable, varying its envelope *decreases* engagement relative to keeping its envelope consistent over time. Meanwhile, when the content of the message is expected to be of little value, varying its envelope *increases* relative engagement.

## Study 2

Study 1 demonstrated that people learn to associate the envelope surrounding digital information interventions with the expected utility value of the message content. However, Study 1 was a controlled online experiment in which participants had a financial incentive to pay close attention to the simulated email subject lines. Thus, in Study 2, we test the impact of varying the digital envelope of a real multiple-round information intervention in a large-scale field experiment (*N* = 199,162).

### Methods

#### Experimental design and setting

In a pre-registered field experiment (https://osf.io/r2j5a), we worked with a large provider of massive open online courses (MOOCs) that sent automated personalized emails to active enrolled students every week. Email content varied each week depending on a student’s progress the prior week. For instance, a student who completed a course activity one week might receive an email the subsequent week congratulating them on their progress and encouraging them to keep a consistent learning schedule. Alternatively, a student who was inactive one week might receive an email encouraging them to make more progress by providing learning tips and upcoming deadlines. All emails were sent from the MOOC provider and included one of the following subject lines:

1. Check out your progress
2. Make every week count
3. New week, new start!
4. Your learning summary

As shown in Table 2, we randomly assigned all active enrolled students to receive one of the four subject lines for their first weekly email update. Then, in order to evaluate the effect of varying the envelope of this multiple-round information intervention, we randomly assigned students to receive either the same subject line each week or a different subject line each week. This design allowed us to isolate the impact of keeping the envelope (subject line) consistent while controlling for any underlying variation in the efficacy of the different subject lines.

**Table 2:** Study 2 design.

		Subject line by week		
Starting subject line	Condition	Week 1	Week 2	Week 3
1	Same subject line	Check out your progress	Check out your progress	Check out your progress
	Varying subject line	Check out your progress	Make every week count	New week, new start!
2	Same subject line	Make every week count	Make every week count	Make every week count
	Varying subject line	Make every week count	New week, new start!	Your learning summary
3	Same subject line	New week, new start!	New week, new start!	New week, new start!
	Varying subject line	New week, new start!	Your learning summary	Check out your progress
4	Same subject line	Your learning summary	Your learning summary	Your learning summary
	Varying subject line	Your learning summary	Check out your progress	Make every week count

#### Sample selection

The sample universe for Study 2 was all students who were actively or newly enrolled in a course from August 3, 2020, the first week during which the MOOC provider began to send weekly update emails, to August 24, 2020. Participants were not required to consent as this study was conducted by the MOOC provider as part of their internal quality improvement processes and the research team only had access to de-identified and anonymized data at the conclusion of the experiment. This study, as well as Study 3, was determined to not constitute human subjects research by Harvard University’s Committee on the Use of Human Subjects (IRB20-0784).

The MOOC provider defined “active” students as those who had completed any course activity in the two weeks prior to the email send date. All students in email-eligible courses (as defined by the MOOC provider) received weekly update emails every Monday until they completed their course or until they were inactive for two consecutive weeks. Because course enrollment occurred on a rolling basis, randomization was also conducted on a weekly basis. The first randomization cohort included all active and newly enrolled students. Thereafter, randomization occurred every Monday for all students who had newly enrolled in an email-eligible course the prior week. Randomization was conducted by the MOOC provider through their digital platform.

In total, 1,579,302 students were randomized across four cohorts (weeks), beginning August 3, 2020. Table 3 shows the exclusion criteria imposed to construct our analytic universe. We exclude the first cohort (*N* = 404,764) from analysis due to an engineering error that led approximately 75% of the students assigned to one of the varying subject line conditions to instead receive the same subject lines in sequential weeks. This error was corrected after the first cohort and did not affect any of the three cohorts included in the analytic universe.

**Table 3:** Study 2 sample construction.

	N
Randomized universe	1,579,302
Cohort 1	404,764
Assigned to control group	234,621
Not active in week 1	735,247
Did not receive any emails	5,508
Final analytic universe	199,162

As specified in our pre-registered analysis plan, we further narrow our analytic universe as follows. First, we exclude 234,621 students who were assigned to a pure control group that did not receive any communication. This group was included at the request of the MOOC provider in order to evaluate the impact of the weekly emails, which is not the focus of the present research. Next, we exclude 735,247 students who were not active in the first week they received an email, balanced evenly across treatment conditions (χ^2^(7) = 11.84, *p* = .11) and pooled treatment conditions (χ^2^(1) = 0.82, *p* = .36). By design, students stopped receiving weekly update emails after two consecutive weeks of inactivity in their enrolled course. Thus, in order to maximize the length of time for which we could observe our outcome of interest and generate unbiased treatment effect estimates, we only include students who were active in the first week of the intervention and, thus, were guaranteed to receive a subsequent two emails. Finally, we exclude 5,508 students who were part of the experimental universe but did not receive any weekly update emails, again balanced evenly across treatment conditions (χ^2^(7) = 10.11, *p* = .18) and pooled treatment conditions (χ^2^(1) = 0.00, *p* = .95). Our final analytic universe consists of 199,162 students who received emails from August 10 to September 7, 2020. Fifty-nine percent received three emails, as intended.

#### Analysis

Our pre-registered primary outcome was engagement with the weekly emails, defined as open rates, over a three-week period. In an intent-to-treat analysis, we evaluate the average treatment effect of condition assignment on email open rates in weeks 2 and 3 of the experiment via a covariate-adjusted logistic model. We also evaluate differential trends in open rates over time, by condition assignment, via a covariate-adjusted interaction model. All models control for course difficulty, course topic area, type of enrollment, geographic location, and randomization cohort. The SOM provides additional detail on analysis models and methods.

We predicted that open rates for the first weekly email would be similar across conditions, and that engagement would decrease over time in all conditions. But we hypothesized that varying the subject line would slow this anticipated decline in engagement. If this was the case, we would expect to see higher average open rates for the second and third weekly emails in the varying subject line conditions than in the same subject line conditions.

We did not hypothesize that changing the subject line would affect course progress or engagement both because we were underpowered to detect such a second stage effect and because this relationship would ultimately depend on the efficacy of the emails themselves—which were not designed as part of the current research. Even if the emails were effective, with low expected open rates across all conditions, we did not anticipate having the statistical power to detect downstream effects on course progress. As predicted, we find no statistically reliable effects on course engagement (see SOM).

### Results and discussion

**Fig 2:**
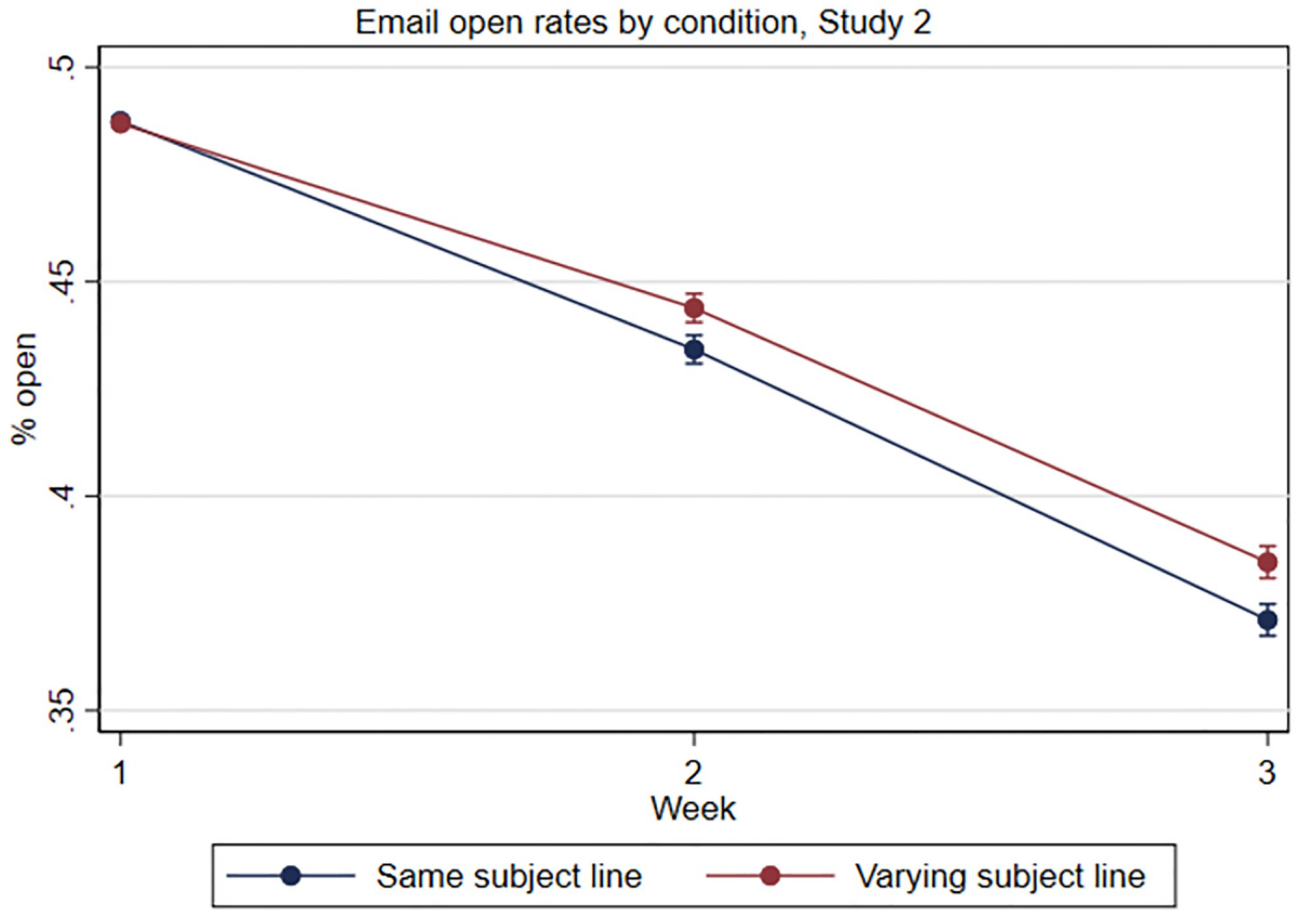
Study 2, email open rates by week and condition assignment.

We find that students assigned to the varying subject line conditions were an average of 1.1 percentage points (pp) or 2.8% more likely to open the emails in weeks 2 and 3 (*M*_*T*_ = 39.8%, *SE* = 0.14) than students who were assigned to receive the same subject line each week (*M*_*C*_ = 40.9%, *SE* = 0.14, *p* < .001). In an interaction model, we find that the effect of time on open rates differs significantly by pooled condition assignment (χ^2^(2) = 26.07, *p* < .001; see Fig 2).

In week 2, the marginal effect of assignment to the varying subject line condition was 1.0pp (*SE* = 0.24, *p* < .001): 43.4% of students assigned to the same subject line condition opened the week 2 email compared to 44.4% of students assigned to the varying subject line condition. To contextualize this effect, the difference between the best and worst performing subject lines in week 2 among students in the same subject line condition was 5.8pp. Thus, the marginal effect of assignment to the varying subject line condition was about 17% of this gap. In week 3, the marginal effect of assignment to the varying subject line condition was 1.3pp (*SE* = 0.27, *p* < .001): 37.1% of students assigned to the same subject line condition opened the week 3 email compared to 38.4% of students assigned to the varying subject line condition. The difference between the best and worst performing subject lines in week 3 among students in the same subject line condition was 4.6pp, so the marginal effect of assignment to the varying subject line condition was about 28% of this gap.

Overall, these findings suggest that varying the subject line of a relatively low-value weekly email increases marginal engagement compared to keeping the subject line the same each week. While engagement with the weekly emails declined for all students over time, varying the subject line each week slowed this decline over a three-week period.

## Study 3

Study 2 tested the impact of varying the digital envelope of a real multiple-round information intervention on engagement over time. However, one important limitation of Study 2 is that the analytic universe was limited to students who were active in their course in the first week of the experiment due to the criteria the MOOC provider used to determine eligibility for the weekly emails. This population had a higher baseline propensity to engage with the emails: on average, 47% of students who were active in their course in the first week opened the first email compared to just 25% of inactive students. It is unclear whether varying the subject line of the weekly email would have the same impact with students who were less likely to engage with the message in the first place. Study 3 addresses this limitation in a second field experiment (*N* = 239,125), conducted in the same context as Study 2, but with a broader and more generalizable sample.

### Methods

#### Experimental design and setting

In Study 3, which was pre-registered (https://osf.io/zsqmt), we worked with the same MOOC provider as in Study 2. However, the MOOC provider relaxed the eligibility criteria for receiving an email such that all newly enrolled students received weekly emails for three consecutive weeks regardless of course activity level. This allowed us to construct an experimental universe comprised of all new students, rather than just active students as was the case in Study 2.

The experimental design of Study 3 was identical to that of Study 2 (see Table 2), but the MOOC provider tested four new subject lines:

1. Check out your progress
2. Weekly update in [course name]
3. Your learning insights in [course name]
4. Learning summary: Week of [date]

#### Sample selection

The sample universe for Study 3 was 579,936 newly enrolled students from December 14, 2020 to January 4, 2021. As in Study 2, participants were not required to consent as this study was conducted by the MOOC provider as part of their internal quality improvement processes and the research team only had access to de-identified and anonymized data at the conclusion of the experiment.

In this study, all newly enrolled students in email-eligible courses (as defined by the MOOC provider) received weekly update emails every Monday for the first three weeks of their enrollment. Thereafter, they continued receiving weekly update emails until they completed their course or until they were inactive for one week. Because course enrollment occurred on a rolling basis, randomization was also conducted on a weekly basis. As in Study 2, randomization was conducted by the MOOC provider through their digital platform and occurred every Monday for all students who had newly enrolled in an email-eligible course the prior week.

In total, Study 3 included three randomized cohorts (weeks), beginning December 21, 2020. As specified in our pre-registered analysis plan, we exclude 340,811 students who were assigned to a set of separate conditions that were included at the request of the MOOC provider in order to evaluate an unrelated research question. Our final analytic universe consists of 239,125 students who received emails from December 21, 2020 to January 18, 2021, 86% of who received three emails as intended.

#### Analysis

As in Study 2, our primary outcome was engagement with the weekly emails, defined as open rates, over a three-week period. Because the sample included all students, regardless of course activity, we expected that average open rates would be significantly lower than in Study 2, but that varying the subject line of the weekly emails would still slow the natural decline in engagement over time.

The analysis methods are the same in Study 2. We first evaluate the average treatment effect of condition assignment on email open rates in weeks 2 and 3 of the experiment using a covariate-adjusted logistic model, and then use an interaction model to evaluate differential trends in open rates over time, by condition assignment. All models control for course difficulty, course topic area, type of enrollment, geographic location, and randomization cohort.

As described in Study 2, we did not hypothesize that changing the subject line would affect course progress. And, as anticipated, we again find no statistically reliable effects on course progress (see SOM).

### Results and discussion

**Fig 3:**
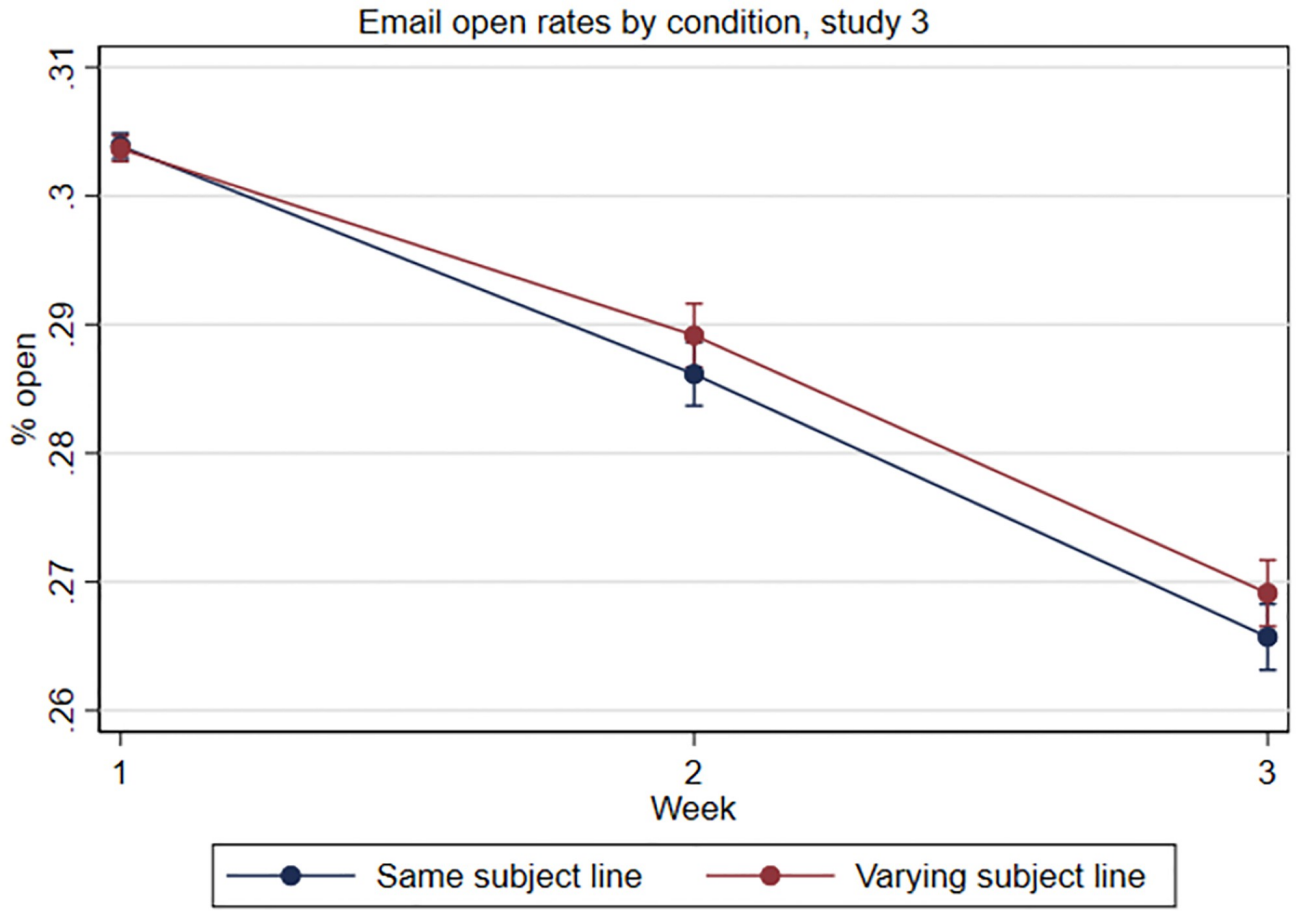
Study 3, email open rates by week and condition assignment.

As predicted, we find that the average open rate for the first weekly email in Study 3 was significantly lower than in Study 2: 30% versus 47%. Still, as shown in Fig 3, we find that students assigned to receive a different subject line each week were an average of 0.3 pp (1.2%) more likely to open the emails in weeks 2 and 3 (*M*_*T*_ = 27.5%, *SE* = 0.10, *p* = .02) than students who received the same subject line each week (*M*_*C*_ = 27.8%, *SE* = 0.10). In an interaction model, the differential effect of condition assignment by time is trending toward significance (χ^2^(2) = 3.65, *p* = .16; see Fig 3) suggesting that, again, varying the subject line of the weekly emails slowed the natural decline in engagement over time.

In week 2, the marginal effect of assignment to the varying subject line condition was 0.3pp (*SE* = 0.18, *p* = .09): 28.6% of students assigned to the same subject line condition opened the week 2 email compared to 28.9% of students assigned to the varying subject line condition. As in Study 2, we can contextualize this effect by examining the difference between the best and worst performing subject lines in week 2 among students in the same subject line condition. Here, this difference was 1.4pp, and so the marginal effect of assignment to the varying subject line condition was about 21% of this gap. In week 3, the marginal effect of assignment to the varying subject line condition was again 0.3pp (*SE* = .19, *p* = .07): 26.6% of students assigned to the same subject line condition opened the week 3 email compared to 26.9% of students assigned to the varying subject line condition. The difference between the best and worst performing subject lines in week 3 among students in the same subject line condition was 1.1pp, so the marginal effect of assignment to the varying subject line condition was about 27% of this gap. Thus, while the absolute magnitude of the effect of assignment to the varying subject line conditions found in Study 3 was smaller than in Study 2, the effect sizes were comparable when evaluated in the context of overall subject line performance in each respective study.

In a fixed effects meta-analysis, we calculate the weighted average treatment effect across Studies 2 and 3. We find that, on average, assignment to the variable subject line condition increased email open rates in weeks 2 and 3 by 0.7 pp (*p* < .001, 95% CI [0.1, 1.5]).

In a subsequent online experiment (*N* = 527) we assessed people’s expectations about the value of the weekly emails sent in Studies 2 and 3 (see SOM for detailed procedures and methods). After reading a description of the MOOC provider’s email program and statistics on average engagement, 62% of participants predicted that the emails would be of little value to them. Taken together, Studies 2 and 3 thus demonstrate that varying the subject line of a repeated digital information intervention can lead to small gains in engagement when the enclosed information is expected to be relatively low value.

## General discussion

Across three studies, we show that engagement with multiple-round digital information interventions is shaped by the envelope surrounding the information. In line with a value utility theory framework, people are less likely to engage with information that they expect to be of low value. In multiple-round interventions, expectations about the value of any given round’s information are informed by experiences with prior rounds. When people encounter the same envelope repeatedly over time, they learn to recognize and associate it with the actual or perceived value of its enclosed information, which then shapes their decision to engage with it in the future.

This research has implications for researchers and policymakers delivering multiple-round digital information interventions in a wide range of contexts, such as those discussed in the introduction. It also offers guidance for firms that routinely communicate with consumers. Firms that deliver high-value information repeatedly over time could increase engagement by using consistent (as opposed to varied) envelopes for each successive round, whereas firms delivering low-value information may be able to increase engagement by using varied (as opposed to consistent) envelopes over time.

The field experiments reported in studies 2 and 3 test our model for low-value messages, finding that for these messages varied envelopes generate more engagement than consistent envelopes. Study 1 reported an online experiment finding the converse: for high-value messages consistent envelopes dominate varied envelopes. Future research should replicate the Study 1 findings regarding high-value messages, but using field experimental designs similar to studies 2 and 3. Relatedly, future research should study, descriptively, whether firms tend to communicate in ways consistent with the model we propose in this manuscript. For example, do firms delivering monthly bills that are relatively consequential (e.g., credit card, mortgage, or utility bills) use consistent envelopes? Conversely, do firms delivering marketing appeals that are relatively inconsequential (e.g., product sales, fundraising appeals) use varied envelopes?

This research also has strategic and ethical implications for communicators. Varying the envelope for low-value messages increases engagement in part by interfering with a recipient’s ability to predict what the envelope contains. In a sense, this sneaks the message into a recipient’s attention by introducing uncertainty about the value of the content, or even curiosity [20]. But in so doing, this strategy is potentially welfare-reducing for recipients if they would otherwise have avoided the message [21, 22]. Future research should further explore and quantify this tradeoff.

## Supporting information

S1 File (DOCX)
