# Supplementary material for: Signals of value drive engagement with multi-round information interventions
Source: PLoS One. 2022 Oct 25;17(10):e0276072. doi: 10.1371/journal.pone.0276072 (PMC9595537; doi:10.1371/journal.pone.0276072)
Supplement: S1 File — (DOCX) [file pone.0276072.s001.docx]

Supporting Information for

Signals of value drive engagement with multi-round information interventions

Jessica Lasky-Fink*

University of California, Berkeley

Todd Rogers

Harvard Kennedy School

***Corresponding author:** jlaskyfink@berkeley.edu

**Contents**

[Supplemental Methods 3](#_Toc92446146)

[Study 1 3](#_Toc92446147)

[Study 2 4](#_Toc92446148)

[Study 3 12](#_Toc92446149)

[Supplemental Studies 17](#_Toc92446150)

[Study S1 17](#_Toc92446151)

# **Supplemental Methods**

## **Study 1**

***Procedure***

After passing the attention check, all participants were given the following instructions:

*In the questions that follow, we will show you email subject lines that you might receive in your inbox. You must choose which email to open. Each email is associated with a $0.50, $0.05, or $0 bonus. After you make your choice, we will show you your bonus amount, as well as the amount you would have received if you had chosen the other email.*

Participants then went through the study procedures presented in the main manuscript.

***Analysis***

We evaluated the differential effect of assignment to the same subject line condition by the value of the enclosed information in the following interaction model:

1. $Y_{i4}=\alpha+\beta_{1}{highvalue}_{i}+\beta_{2}{subjectline}_{i}+\beta_{3}highvalueX{subjectline}_{i}\boldsymbol{+X}+\varepsilon_{i}$

where $Y_{i4}$ is an indicator for whether participant *i* opened the focal message in the fourth stage; *highvalue* is a binary indicator for assignment to one of the high value conditions (i.e., $0.50 bonus); *subjectline* is a binary indicator for assignment to one of the “same” subject line conditions; and ***X*** is a vector of participant-level covariates including gender, age, college education, and party affiliation. The coefficient, $\beta_{3}$, is interpreted as the differential effect of a consistent envelope (i.e., subject lines) by the value of the enclosed information.

***Results***

**Table S1.** Study 1 results: Choice of focal subject line in fourth stage

|  | (1) |
| --- | --- |
| VARIABLES | Chose focal SL in fourth stage |
|  |  |
| High value SL | 0.140*** |
|  | (0.042) |
| Same SL | -0.313*** |
|  | (0.041) |
| High value X Same SL | 0.547*** |
|  | (0.059) |
| Age | 0.002 |
|  | (0.001) |
| Female | 0.056 |
|  | (0.030) |
| Other gender | -0.283 |
|  | (0.251) |
| College educated | -0.041 |
|  | (0.031) |
| Republican | -0.020 |
|  | (0.036) |
| Independent | 0.044 |
|  | (0.037) |
| Other party affiliation | -0.040 |
|  | (0.090) |
|  |  |
| Observations | 863 |
| R-squared | 0.264 |
| Mean for low value, same SL | 0.480 |

Notes: Logistic estimates of equation (1). Outcome is binary indicator for whether participant chose the focal subject line associated with their condition assignment in the fourth stage of the study. Standard errors in parentheses. *** p<0.001, ** p<0.01, * p<0.05

## **Study 2**

***Study Setting***

Study 2 was implemented in partnership with a large provider of Massive Open Online Courses (MOOCs). The MOOC provider offers hundreds of online courses of varying lengths and degrees of difficulties. There are four channels through which a student can enroll in a course: paid plans, financial aid plans, free plans, or auditing. If a student enrolls via a paid or financial aid plan, they can receive a formal certificate and credit if they complete the course. If a student enrolls via a free plan, they have access to all course materials and activities, can submit required assessments, and can receive a final grade, but they cannot receive a certificate for completion. And if a student audits a course, they can access most of the course materials, but cannot submit all assignments, complete the course, receive a course grade, or receive a certificate.

As of Fall 2020, approximately 1.1 million new students were enrolling via free or audit plans each week. Approximately 130,000 new students were enrolling via financial aid or paid plans each week. Students enrolled via paid or financial aid plans are, on average, more engaged. Approximately 54% of students enrolled via a paid plan complete their course compared to about 30% of students enrolled via a financial aid plan and 5% of students on free plans.

In August 2020, the MOOC provider began sending weekly update emails to all newly enrolled students in certain courses. If a student was enrolled in more than one email-eligible course, the weekly emails were only sent for their most recent course enrollment. The weekly emails offered personalized information on a student’s course progress to date, next steps for continued learning, and weekly learning goals. The goal of the emails was to keep students on track in their learning, and ultimately to increase course engagement and completion.

***Study Design***

The randomization was conducted by the MOOC provider on a weekly basis. Each Monday during the experiment, all students who had newly enrolled in an email-eligible course in the prior seven days were randomized with equal probability to one of nine conditions, as shown in Table S2.

Students in the control condition (#9) did not receive weekly update emails. This condition was included at the request of the MOOC provider to answer an unrelated research question. Because we had four different subject lines, the randomization was factorialized by subject line. All students were randomly assigned to receive one of the four subject lines the first week. Then, students in one of the “same” subject line conditions (conditions 1, 3, 5, 7) continued to receive the same subject line in all subsequent weeks. Meanwhile, students in one of the “varying” subject line conditions (conditions 2, 4, 6, 8) received a different subject line each week. For these conditions, the order of the subject lines rotated in a consistent manner such that students who started with subject line A then received subject line B the subsequent week, followed by subject line C, and so on.

**Table S2.** Study 2 design

|  |  | Subject Line | | |
| --- | --- | --- | --- | --- |
|  | Condition | Week 1 | Week 2 | Week 3 |
| Treatment | 1. A-same | A | A | A |
|  | 2. A-varying |  | B | C |
|  | 3. B-same | B | B | B |
|  | 4. B-varying |  | C | D |
|  | 5. C-same | C | C | C |
|  | 6. C-varying |  | D | A |
|  | 7. D-same | D | D | D |
|  | 8. D-varying |  | A | B |
| Control | 9. Control | None | | |

***Data and Outcomes***

Our primary outcome of interest was engagement with each weekly email, defined as email opens. Email opens were tracked by the MOOC provider. We defined our outcome as an open in the seven days (inclusive) after receiving the email. See Table S3. All data came from routinely collected administrative data and meta-data provided by the MOOC provider.

The goal of the weekly emails was to increase course engagement, persistence, and completion. However, we did not pre-register outcomes of course progress or engagement for two reasons. First, for the weekly emails to affect these outcomes, the emails themselves would have to be effective. The emails were not designed as part of the current research, nor had their efficacy been evaluated prior to conducting the present experiment. Second, even if the emails themselves were effective, we determined ex ante that we would be underpowered to detect a second stage effect in this intervention due to anticipated low email open rates and effect sizes. Nevertheless, as an exploratory analysis we report average treatment effects on two measures of course engagement in Table S8: course activity and course components completed. The former is defined as a binary student-level indicator in which a 1 reflects any activity taken in a given week. The latter is a student-level continuous measure of the number of course items or activities (e.g., lectures, assignments, quizzes) completed in a given week.

**Table S3.** Study 2 timeline

| Date email received | Outcome period | Cohort 1 | Cohort 2 | Cohort 3 |
| --- | --- | --- | --- | --- |
| Aug. 10, 2020 | Aug. 10 – Aug. 17 | X |  |  |
| Aug. 17, 2020 | Aug. 17 – Aug. 24 | X | X |  |
| Aug. 24, 2020 | Aug. 24 – Aug. 31 | X | X | X |
| Aug. 31, 2020 | Aug. 31 – Sept. 7 |  | X | X |
| Sept. 7, 2020 | Sept. 7 – Sept. 14 |  |  | X |

***Analysis***

To evaluate the main effect of varying the subject line on email open rates, we first evaluated the following logistic model for weeks 2-3 of the intervention period:

1. $Y_{ij}=\alpha+\beta_{1}{treatpooled}_{ij}+\beta_{2}{week}_{j}+\beta_{3}{week1open}_{i1}{+ \beta}_{4}{cohort}_{ij}+X_{i}+\delta+\varepsilon_{ij}$

where $Y_{ij}$ reflects whether student *i* in week *j*^[[1]](#footnote-1)^ opened the weekly update email; ${treatpooled}_{ij}$ is a pooled indicator for experimental condition assignment for student *i* in intervention week *j*; ${week}_{j}$ is fixed effects for intervention week; ${week1open}_{i1}$ is a binary indicator reflecting whether student *i* opened the first weekly email update; ${cohort}_{ij}$ reflects the randomization cohort for student *i*; $X_{i}$ is a vector of student-level covariates described below; and $\delta$ is a vector of course-level covariates, including difficulty level and subject domain. Robust standard errors were clustered by student.

Our coefficient of interest in this model, $\beta_{1}$, reflects the average effect of subject line variability on email open rates during weeks 2 and 3 of the experiment. By construction, students who did not receive an email in weeks 2 or 3 are excluded from this analysis.^[[2]](#footnote-2)^

The treatment indicator was pooled such that students assigned to any variable subject line condition (#2, 4, 6, 8) were analyzed as a single condition relative to students assigned to any of the same subject line conditions (#1, 3, 5, 7). Because past email open behavior is strongly predictive of future behavior, we also controlled for whether a student opened the first email update they received. The first email update was sent after randomization, but prior to the start of the same vs. varying subject line treatment, which began in week 2 for each cohort.

Next, to examine the effect of varying the subject line on email open rates over time, we also evaluated the following logistic interaction model:

1. $Y_{ij}=\alpha+\beta_{1}{treatpooledXweek}_{ij}+\beta_{2}{treatpooled}_{i}+\beta_{3}{week}_{j}+\beta_{4}{week1open}_{i1}+{+ \beta}_{5}{cohort}_{ij}+X_{i}+\delta+\varepsilon_{ij}$

where ${treatpooledXweek}_{ij}$ reflects the interaction of the binary indicator for student *i*’s condition assignment by intervention week *j*; and the rest of the variables are as described in model (2). Our coefficient of interest in this model, $\beta_{1}$, can be interpreted as the differential effect of varying the subject line on email open rates over time.

Student-level covariates included country of residence (USA or non-USA) and type of enrollment (free, financial aid, paid, or audit; see Study Context). Students are not required to provide demographic information when enrolling in a course. Although student-level demographics including gender and education level are imputed by learning platform algorithms, up to 50% of students are missing covariates that cannot be imputed. Thus, student demographics were not included in our primary specifications. However, as a robustness check, we also evaluated equations (2) and (3) including student gender and education level, as well as indicators for missing values of each demographic variable.

***Results***

Of the 199,162 students in the final analytic universe, 62% received at least three emails as intended. See Table S4. Tables S5 – S7 present estimates from equations (2) and (3) for Study 2.

**Table S4.** Study 2, number of emails received

| Number of emails received | N | % |
| --- | --- | --- |
| 1 | 42,024 | 21.1% |
| 2 | 38,839 | 19.5% |
| 3+ | 118,299 | 59.4% |

**Table S5.** Study 2, regression-adjusted average open rates by condition, by week

| Intervention week | % students who opened email | |
| --- | --- | --- |
|  | Same subject line | Varying subject line |
| 1 | 48.7% | 48.7% |
| 2 | 43.4% | 44.4% |
| 3 | 37.1% | 38.5% |

**Table S6.** Study 2, logistic estimates of equation (2)

|  | (1) | (2) |
| --- | --- | --- |
| VARIABLES | Email open, weeks 2-3 | Email open, weeks 2-3 |
|  |  |  |
| Varying subject line | 0.056*** | 0.057*** |
|  | (0.010) | (0.010) |
| Intervention week 3 | -0.310*** | -0.308*** |
|  | (0.007) | (0.007) |
| Email opened in week 1 | 1.643*** | 1.631*** |
|  | (0.010) | (0.010) |
| Enrollment plan: Financial aid | 0.341*** | 0.307*** |
|  | (0.020) | (0.021) |
| Enrollment plan: Free | 0.155*** | 0.151*** |
|  | (0.015) | (0.015) |
| Enrollment plan: Free trial | 0.270** | 0.257** |
|  | (0.084) | (0.085) |
| Enrollment plan: Paid | 0.482*** | 0.457*** |
|  | (0.017) | (0.017) |
| Location: USA | 0.155*** | 0.131*** |
|  | (0.012) | (0.012) |
| Randomization cohort 2 | -0.060*** | -0.058*** |
|  | (0.012) | (0.012) |
| Randomization cohort 3 | -0.015 | -0.013 |
|  | (0.012) | (0.012) |
| Female |  | 0.031* |
|  |  | (0.013) |
| Education level: Less than HS diploma |  | -0.202*** |
|  |  | (0.057) |
| Education level: HS diploma |  | -0.156*** |
|  |  | (0.040) |
| Education level: Some college |  | -0.134*** |
|  |  | (0.037) |
| Education level: BA/BS |  | -0.016 |
|  |  | (0.034) |
| Education level: MA/MS |  | 0.040 |
|  |  | (0.035) |
| Education level: Professional degree |  | 0.097* |
|  |  | (0.047) |
| Education level: Doctorate |  | 0.174*** |
|  |  | (0.049) |
|  |  |  |
| Observations | 275,980 | 275,980 |
| Clusters | 158,406 | 158,406 |
| Mean open rate, same SL condition | 0.398 | 0.398 |

Notes: Column (1) reports logistic estimates of equation (2); Column (2) reports logistic estimates of equation (2) with additional demographic control variables. Outcome is a binary indicator for whether the weekly email was opened. Number of clusters is less than total N in analytic universe because 40,756 students in the analytic universe did not receive emails in weeks 2 or 3. Both models include controls for course domain and course difficulty level. Column (2) also includes controls for missing gender and missing education level. Reference groups: same subject line condition; intervention week 2; week 1 email not opened; enrollment plan = audit; location non-US; randomization cohort 1; male; education = associate degree. Standard errors in parentheses. *** p<0.001, ** p<0.01, * p<0.05

**Table S7.** Study 2, logistic estimates of equation (3)

|  | (1) | (2) |
| --- | --- | --- |
| VARIABLES | Email open | Email open |
|  |  |  |
| Varying subject line | -0.004 | -0.004 |
|  | (0.006) | (0.006) |
| Intervention week 2 | -0.375*** | -0.374*** |
|  | (0.013) | (0.013) |
| Intervention week 3 | -0.816*** | -0.813*** |
|  | (0.015) | (0.015) |
| Varying subject line X week 2 | 0.071*** | 0.071*** |
|  | (0.019) | (0.019) |
| Varying subject line X week 3 | 0.097*** | 0.098*** |
|  | (0.021) | (0.021) |
| Email opened in week 1 | 3.108*** | 3.099*** |
|  | (0.009) | (0.009) |
| Enrollment plan: Financial aid | 0.294*** | 0.267*** |
|  | (0.017) | (0.018) |
| Enrollment plan: Free | 0.132*** | 0.129*** |
|  | (0.013) | (0.013) |
| Enrollment plan: Free trial | 0.230*** | 0.218*** |
|  | (0.025) | (0.025) |
| Enrollment plan: Paid | 0.414*** | 0.392*** |
|  | (0.014) | (0.015) |
| Location: USA | 0.123*** | 0.103*** |
|  | (0.010) | (0.010) |
| Randomization cohort 2 | -0.052*** | -0.051*** |
|  | (0.009) | (0.009) |
| Randomization cohort 3 | 0.018 | 0.021* |
|  | (0.011) | (0.011) |
| Female |  | 0.025* |
|  |  | (0.011) |
| Education level: Less than HS diploma |  | -0.152** |
|  |  | (0.049) |
| Education level: HS diploma |  | -0.117*** |
|  |  | (0.034) |
| Education level: Some college |  | -0.112*** |
|  |  | (0.032) |
| Education level: BA/BS |  | -0.006 |
|  |  | (0.029) |
| Education level: Professional degree |  | 0.084* |
|  |  | (0.041) |
| Education level: MA/MS |  | 0.046 |
|  |  | (0.030) |
| Education level: Doctorate |  | 0.154*** |
|  |  | (0.041) |
|  |  |  |
| Observations | 468,806 | 468,806 |
| Clusters | 199,162 | 199,162 |
| Week 1, same SL mean | 0.487 | 0.487 |

Notes: Column (1) reports logistic estimates of equation (3); Column (2) reports logistic estimates of equation (3) with additional demographic control variables. Outcome is a binary indicator for whether the weekly email was opened. Both models include controls for course domain and course difficulty level. Column (2) also includes controls for missing gender and missing education level. Reference groups: same subject line condition; intervention week 1; week 1 email not opened; enrollment plan = audit; location non-US; randomization cohort 1; male; education = associate degree. Standard errors in parentheses. *** p<0.001, ** p<0.01, * p<0.05

**Table S8.** Study 2, effect of condition assignment on course engagement

|  | (1) | (2) |
| --- | --- | --- |
| VARIABLES | Active in course, wk 2-3 | Course items completed, wk 2-3 |
|  |  |  |
| Varying subject line | 0.004 | -0.037 |
|  | (0.009) | (0.036) |
| Intervention week 3 | -0.681*** | -2.248*** |
|  | (0.006) | (0.027) |
| Email opened in week 1 | 0.203*** | 0.721*** |
|  | (0.009) | (0.037) |
| Enrollment plan: Financial aid | 1.441*** | 5.151*** |
|  | (0.019) | (0.081) |
| Enrollment plan: Free | 0.600*** | 2.026*** |
|  | (0.015) | (0.043) |
| Enrollment plan: Free trial | -1.510*** | -2.045*** |
|  | (0.056) | (0.083) |
| Enrollment plan: Paid | 1.666*** | 5.373*** |
|  | (0.017) | (0.061) |
| Location: USA | 0.003 | 0.134** |
|  | (0.012) | (0.045) |
| Randomization cohort 2 | -0.048*** | -0.158*** |
|  | (0.011) | (0.046) |
| Randomization cohort 3 | -0.218*** | -1.078*** |
|  | (0.012) | (0.043) |
|  |  |  |
| Observations | 297,993 | 297,993 |
| Clusters | 163,782 | 163,782 |
| R-squared |  | 0.108 |
| Mean open rate, same SL condition | 0.446 | 4.617 |

Notes: Column (1) reports logistic estimates of the impact of condition assignment on a binary indicator for course activity, where 1 reflects any course activity completed in a given week. Column (2) reports OLS estimates of the impact of condition assignment on a continuous measure of course components completed each week. Both models include controls for course domain and course difficulty level. Number of clusters is less than total N in analytic universe because 35,380 students are missing data for intervention weeks 2 and 3. Reference groups: same subject line condition; intervention week 2; week 1 email not opened; enrollment plan = audit; location non-US; randomization cohort 1. Standard errors in parentheses. *** p<0.001, ** p<0.01, * p<0.05

## **Study 3**

***Data and Outcomes***

Study 3 was conducted from December 2020 to January 2021, and included three randomization cohorts (weeks). As in Study 2, our primary outcome of interest was engagement with each weekly email, defined as email opens. Email opens were tracked by the MOOC provider. We defined our outcome as an open in the seven days (inclusive) after receiving the email. See Table S9. All data came from routinely collected administrative data and meta-data provided by the MOOC provider.

As in Study 2, we did not pre-register course progress or engagement as outcomes, but in an exploratory analysis we still report average treatment effects on two measures of course engagement in Table S14.

**Table S9.** Study 3 timeline

| Date email received | Outcome period | Cohort 1 | Cohort 2 | Cohort 3 |
| --- | --- | --- | --- | --- |
| Dec. 21, 2020 | Dec. 21 – Dec. 28 | X |  |  |
| Dec. 28, 2020 | Dec. 28 – Jan. 4 | X | X |  |
| Jan. 4, 2021 | Jan. 4 – Jan. 11 | X | X | X |
| Jan. 11, 2021 | Jan. 11 – Jan. 18 |  | X | X |
| Jan. 18, 2021 | Jan. 18 – Jan. 25 |  |  | X |

***Analysis***

The analysis methods for Study 3 were identical to those of Study 2.

***Results***

Of the 239,125 students in the final analytic universe, 86% received at least three emails as intended. See Table S10. Tables S11 – S13 present estimates from equations (2) and (3) for Study 3.

**Table S10.** Study 3, number of emails received

| Number of emails received | N | % |
| --- | --- | --- |
| 1 | 13,000 | 5.4% |
| 2 | 20,750 | 8.7% |
| 3+ | 205,375 | 85.9% |

**Table S11.** Study 3, regression-adjusted average open rates by condition, by week

| Intervention week | % students who opened email | |
| --- | --- | --- |
|  | Same subject line | Varying subject line |
| 1 | 30.4% | 30.4% |
| 2 | 28.6% | 28.9% |
| 3 | 26.6% | 26.9% |

**Table S12.** Study 3, logistic estimates of equation (2)

|  | (1) | (2) |
| --- | --- | --- |
| VARIABLES | Email open, weeks 2-3 | Email open, weeks 2-3 |
|  |  |  |
| Varying subject line | 0.019* | 0.019* |
|  | (0.008) | (0.008) |
| Intervention week 3 | -0.123*** | -0.123*** |
|  | (0.006) | (0.006) |
| Email opened in week 1 | 1.823*** | 1.811*** |
|  | (0.009) | (0.009) |
| Enrollment plan: Financial aid | 0.377*** | 0.312*** |
|  | (0.022) | (0.023) |
| Enrollment plan: Free | 0.048*** | 0.042*** |
|  | (0.011) | (0.011) |
| Enrollment plan: Free trial | 0.318*** | 0.284** |
|  | (0.090) | (0.090) |
| Enrollment plan: Paid | 0.463*** | 0.429*** |
|  | (0.019) | (0.019) |
| Location: USA | 0.287*** | 0.249*** |
|  | (0.010) | (0.011) |
| Randomization cohort 2 | 0.000 | 0.003 |
|  | (0.010) | (0.010) |
| Randomization cohort 3 | -0.020* | -0.019 |
|  | (0.010) | (0.010) |
| Female |  | 0.022 |
|  |  | (0.012) |
| Education level: Less than HS diploma |  | -0.132* |
|  |  | (0.057) |
| Education level: HS diploma |  | -0.101* |
|  |  | (0.043) |
| Education level: Some college |  | -0.137*** |
|  |  | (0.041) |
| Education level: BA/BS |  | 0.016 |
|  |  | (0.037) |
| Education level: Professional degree |  | 0.111* |
|  |  | (0.049) |
| Education level: MA/MS |  | 0.080* |
|  |  | (0.038) |
| Education level: Doctorate |  | 0.200*** |
|  |  | (0.049) |
|  |  |  |
| Observations | 431,500 | 431,500 |
| Clusters | 226,125 | 226,125 |
| Mean open rate, same SL condition | 0.275 | 0.275 |

Notes: Column (1) reports logistic estimates of equation (2); Column (2) reports logistic estimates of equation (2) with additional demographic control variables. Outcome is a binary indicator for whether the weekly email was opened. Number of clusters is less than total N in analytic universe because 13,000 students in the analytic universe are missing data for intervention weeks 2 and 3. Both models include controls for course domain and course difficulty level. Column (2) also includes controls for missing gender and missing education level. Reference groups: same subject line condition; intervention week 2; week 1 email not opened; enrollment plan = audit; location non-US; randomization cohort 1; male; education = associate degree. Standard errors in parentheses. *** p<0.001, ** p<0.01, * p<0.05

**Table S13.** Study 3, logistic estimates of equation (3)

|  | (1) | (2) |
| --- | --- | --- |
| VARIABLES | Email open | Email open |
|  |  |  |
| Varying subject line | -0.002 | -0.002 |
|  | (0.006) | (0.006) |
| Intervention week 2 | -0.139*** | -0.140*** |
|  | (0.012) | (0.012) |
| Intervention week 3 | -0.305*** | -0.306*** |
|  | (0.012) | (0.012) |
| Varying subject line X week 2 | 0.026 | 0.026 |
|  | (0.016) | (0.016) |
| Varying subject line X week 3 | 0.030 | 0.030 |
|  | (0.017) | (0.017) |
| Email opened in week 1 | 3.049*** | 3.041*** |
|  | (0.007) | (0.007) |
| Enrollment plan: Financial aid | 0.328*** | 0.273*** |
|  | (0.020) | (0.020) |
| Enrollment plan: Free | 0.043*** | 0.039*** |
|  | (0.010) | (0.010) |
| Enrollment plan: Free trial | 0.195*** | 0.179*** |
|  | (0.021) | (0.021) |
| Enrollment plan: Paid | 0.408*** | 0.378*** |
|  | (0.017) | (0.017) |
| Location: USA | 0.243*** | 0.211*** |
|  | (0.009) | (0.009) |
| Randomization cohort 2 | -0.001 | 0.002 |
|  | (0.009) | (0.009) |
| Randomization cohort 3 | -0.019* | -0.018* |
|  | (0.009) | (0.009) |
| Female |  | 0.018 |
|  |  | (0.010) |
| Education level: Less than HS diploma |  | -0.113* |
|  |  | (0.049) |
| Education level: HS diploma |  | -0.082* |
|  |  | (0.037) |
| Education level: Some college |  | -0.112** |
|  |  | (0.035) |
| Education level: BA/BS |  | 0.017 |
|  |  | (0.032) |
| Education level: Professional degree |  | 0.097* |
|  |  | (0.043) |
| Education level: MA/MS |  | 0.072* |
|  |  | (0.033) |
| Education level: Doctorate |  | 0.174*** |
|  |  | (0.043) |
|  |  |  |
| Observations | 670,625 | 670,625 |
| Clusters | 239,125 | 239,125 |
| Week 1, same SL | 0.304 | 0.304 |

Notes: Column (1) reports logistic estimates of equation (3); Column (2) reports logistic estimates of equation (3) with additional demographic control variables. Outcome is a binary indicator for whether the weekly email was opened. Both models include controls for course domain and course difficulty level. Column (2) also includes controls for missing gender and missing education level. Reference groups: same subject line condition; intervention week 1; week 1 email not opened; enrollment plan = audit; location non-US; randomization cohort 1; male; education = associate degree. Standard errors in parentheses. *** p<0.001, ** p<0.01, * p<0.05

**Table S14.** Study 2, effect of condition assignment on course engagement

|  | (1) | (2) |
| --- | --- | --- |
| VARIABLES | Active in course, wk 2-3 | Course items completed, wk 2-3 |
|  |  |  |
| Varying subject line | 0.004 | 0.015 |
|  | (0.012) | (0.019) |
| Intervention week 3 | -0.214*** | -0.189*** |
|  | (0.007) | (0.014) |
| Email opened in week 1 | 0.727*** | 0.831*** |
|  | (0.012) | (0.023) |
| Enrollment plan: Financial aid | 1.897*** | 3.586*** |
|  | (0.025) | (0.093) |
| Enrollment plan: Free | 0.408*** | 0.488*** |
|  | (0.018) | (0.019) |
| Enrollment plan: Free trial | 2.152*** | 4.629*** |
|  | (0.084) | (0.476) |
| Enrollment plan: Paid | 2.329*** | 5.110*** |
|  | (0.022) | (0.086) |
| Location: USA | 0.157*** | 0.285*** |
|  | (0.015) | (0.026) |
| Randomization cohort 2 | 0.077*** | 0.003 |
|  | (0.015) | (0.023) |
| Randomization cohort 3 | 0.076*** | 0.021 |
|  | (0.015) | (0.022) |
|  |  |  |
| Observations | 431,500 | 431,500 |
| Clusters | 226,125 | 226,125 |
| R-squared |  | 0.076 |
| Mean open rate, same SL condition | 0.117 | 1.210 |

Notes: Column (1) reports logistic estimates of the impact of condition assignment on a binary indicator for course activity, where 1 reflects any course activity completed in a given week. Column (2) reports OLS estimates of the impact of condition assignment on a continuous measure of course components completed each week. Both models include controls for course domain and course difficulty level. Number of clusters is less than total N in analytic universe because 13,000 students are missing data for intervention weeks 2 and 3. Reference groups: same subject line condition; intervention week 2; week 1 email not opened; enrollment plan = audit; location non-US; randomization cohort 1. Standard errors in parentheses. *** p<0.001, ** p<0.01, * p<0.05

# **Supplemental Studies**

## **Study S1**

***Participants***

Participants were 527 workers (Mage = 39 years; 52% female) on Amazon Mechanical Turk (MTurk) who were recruited to complete a 2-minute online survey for which they were each paid $0.40. Only MTurk workers located in the United States, who had an MTurk approval rating of at least 95%, who had not participated in previous similar studies, who consented to participate, and who passed an initial attention check were eligible to complete the survey.

***Procedure***

All workers who consented to participate and passed the attention check were randomly assigned by the survey platform to one of four experimental conditions:

1. Same subject line, two weeks (*N* = 134)
2. Different subject line, two weeks (*N* = 136)
3. Same subject line, three weeks (*N* = 129)
4. Different subject line, three weeks (*N* = 132)

In the “two weeks” conditions (conditions 1 and 2), participants were given the following prompt:

*Imagine you enroll in a free online course through [MOOC platform].*

*Your first week, you receive an email from [MOOC platform] that encourages you to engage with the course. 30% of students open the email, and 4% click on the link inside.*

*In your second week, you receive another email from the same sender with [the same // a different] subject line.*

*Would you predict that you would find the content in this email:*

- *High value and very useful*
- *Low value and not very useful*

Participants in the “same subject line” condition (condition 1) were told that the second email had the same subject line as the first, while participants in the “different subject line” condition (condition 2) were told that the second email had a different subject line than the first.

In the “three weeks” conditions (conditions 3 and 4), participants were given the following prompt:

*Imagine you enroll in a free online course through [MOOC platform].*

*Your first week, you receive an email from [MOOC platform] that encourages you to engage with the course. 30% of students open the email, and 4% click on the link inside.*

*Your second week, you receive another email from the same sender with [the same // a different] subject line and similar content encouraging you to engage with the course. 30% of students open the email, and 4% click on the link inside.*

*In your third week, you receive another email from the same sender with [the same // a different] subject line.*

*Would you predict that you would find the content in this email:*

- *High value and very useful*
- *Low value and not very useful*

As in the “two weeks” conditions, participants in the “same subject line” condition (condition 3) were told that the second email had the same subject line as the first, while participants in the “different subject line” condition (condition 4) were told that the second email had a different subject line than the first.

***Results***

Overall, 62% of participants across all conditions believed that the final email would be of low value. Among participants assigned to one of the “same subject line” conditions (conditions 1 and 3), 66% predicted the content of the final email would be low value, compared to 58% of participants assigned to one of the “different subject line” conditions (conditions 2 and 4) (χ^2^(1) = 3.54, *p* = .06).

Disaggregating by whether participants were told they received two emails (conditions 1 and 2) or three emails (conditions 3 and 4), we see similar patterns. Among participants in the “two weeks” conditions, 67% of those who were told they received the same subject line both weeks predicted the final email would be relatively low value compared to 56% of those who were told they received a different subject line the second week. Similarly, among participants in the “three weeks” conditions, 65% of those who were told they received the same subject line all three weeks predicted the final email would be relatively low value compared to 60% of those who were told they received a different subject line each week.

1. Randomization cohorts were pooled such that week *j* reflects intervention week, rather than calendar week. [↑](#footnote-ref-1)
2. Students may not have received an email in weeks 2 or 3 if they opted out after receiving their first email, or due to underlying business logic used by the MOOC provider to determine email eligibility each week. [↑](#footnote-ref-2)
